# An alternative and effective method for extracting skeletal organic matrix adapted to the red coral *Corallium rubrum*

**DOI:** 10.1242/bio.059536

**Published:** 2022-10-14

**Authors:** Philippe Ganot, Guillaume Loentgen, Frédéric Marin, Laurent Plasseraud, Denis Allemand, Sylvie Tambutté

**Affiliations:** ^1^ Unité de Recherche sur la Biologie des Coraux Précieux CSM – CHANEL, Centre Scientifique de Monaco, 8 Quai Antoine 1er, MC 98000 Monaco, Principality of Monaco; ^2^ Coral Physiology and biochemistry Laboratory, Marine Biology Department, Centre Scientifique de Monaco, 8 Quai Antoine 1er, MC 98000, Monaco, Principality of Monaco; ^3^ Biogéosciences, UMR CNRS-EPHE 6282, Université de Bourgogne – Franche-Comté, 6, Boulevard Gabriel, bâtiment Gabriel, 21000 DIJON, France; ^4^ ICMUB Institut de Chimie Moléculaire de l'Université de Bourgogne, UMR CNRS 6302, Université de Bourgogne - Franche-Comté, 9, Avenue Alain Savary, bâtiment Mirande, 21000 DIJON, France; ^5^ Centre Scientifique de Monaco, 8 Quai Antoine 1er, MC 98000 Monaco, Principality of Monaco

**Keywords:** Biomineralization, Calcification, Octocorals

## Abstract

Skeleton formation in corals is a biologically controlled process in which an extracellular organic matrix (OM) is entrapped inside the calcified structure. The analysis of OM requires a time-consuming and tedious extraction that includes grinding, demineralization, multiple rinsing and concentration steps. Here we present an alternative and straightforward method for the red coral *Corallium rubrum* that requires little equipment and saves steps. The entire skeleton is directly demineralized to produce a tractable material called ghost, which is further rinsed and melted at 80°C in water. The comparative analysis of the standard and alternative methods by electrophoresis, western blot, and FTIR of *C. rubrum* OM, shows that the ‘alternative OM’ is of higher quality. Advantages and limitations of both methods are discussed.

## INTRODUCTION

Corals are biomineralizing organisms that form composite skeletons made of calcium carbonate and of an organic fraction, the organic matrix (OM). The OM consists of proteins, lipids and sugars and plays major roles in the different steps of the mineralization process, from stabilization of amorphous phases (Falini et al., 2015), to crystal growth and shaping (Tambutté et al., 2008). Studies aiming at characterizing OM properties and composition are consequently key to understand how skeletons form in corals.

An important prerequisite for most investigations on OM relates to the extraction stage since OM is occluded within the mineral phase. From a methodological point of view, this implies several steps, including removal of the tissues, cryogenic grinding of the skeleton into powder, extensive cleaning and rinsing, demineralization, and finally multiple rinsing and OM concentration. Extensive cleaning steps of powder have been mostly used for OM extraction since both endogenous cellular and exogenous contaminants may be trapped in growing biominerals (Ramos-Silva et al., 2013). Arguably, this step discards inter-crystalline macromolecules though it is not yet clear what can be considered as “contaminants” and whether cytoplasmic components (e.g. histones, actin, tubulin) are contaminants or true OM components (Le Roy et al., 2021). In addition, some components essential for biomineralization but sensitive to bleaching agents may be altered or lost (Goffredo et al., 2011) and this loss depends strongly on the particle size of the powder. Finally, grinding into fine powder (admittedly below 30 µm grain size) requires specialized equipment such as a cryo-grinder and/or tedious manual steps. Therefore, this method, currently used for all corals, has its limitations.

**Fig. 1.**
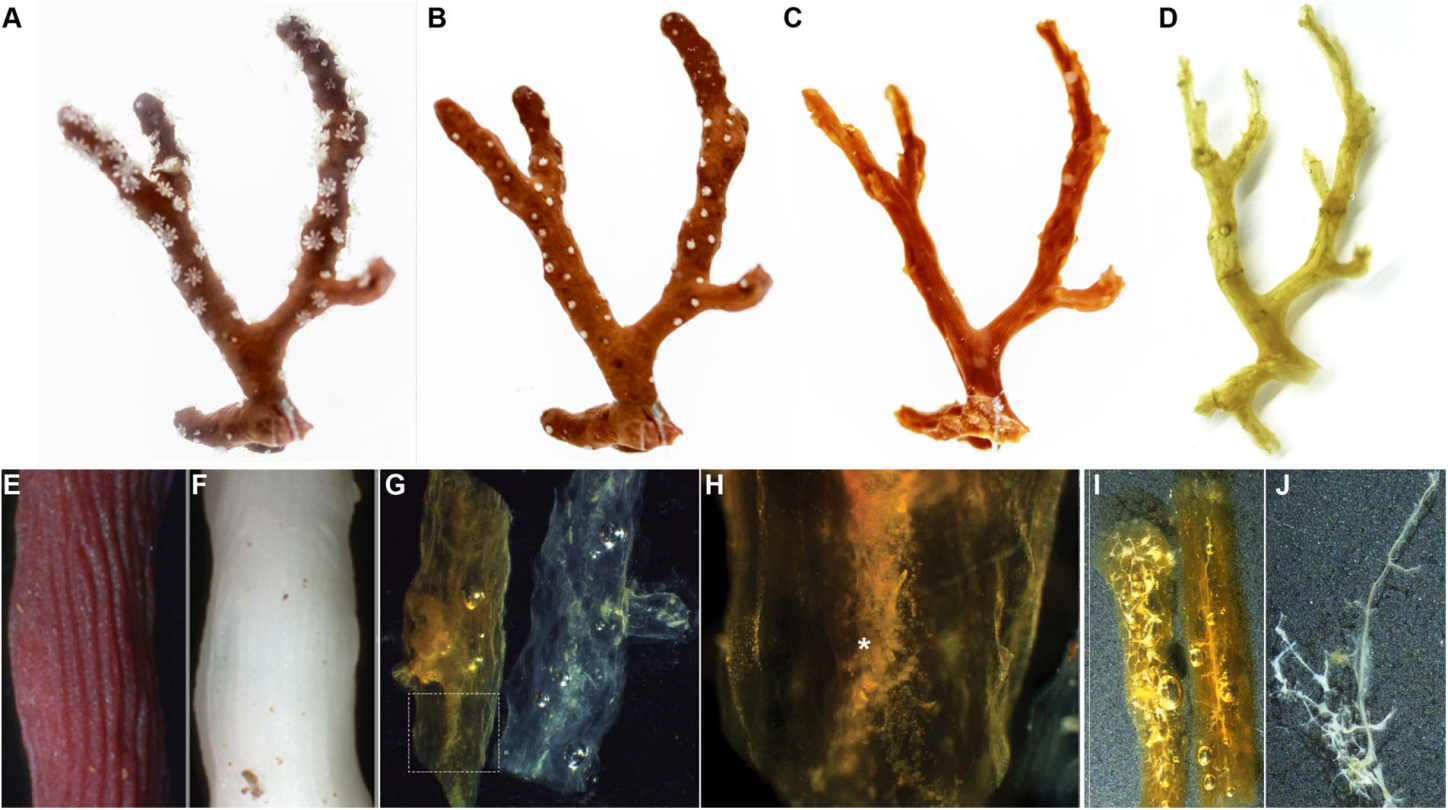
**The *Corallium rubrum* skeleton before and after demineralization (ghost).** (A) *C. rubrum* living colony with extended polyps; (B) the same living colony with retracted polyps; (C) the remaining skeleton of this colony after bleaching; (D) the residual axial skeleton ghost after complete demineralization. (E,F) Focus on a skeleton from a red (E) and a naturally occurring albinos (F) *C. rubrum* colony. (G) Ghosts produced from a red (left) and an albinos colony (right): note the red-orange and the white colours of the ghosts extracted from a red and albinos colony, respectively. (H) Detail of a ghost extracted from a red skeleton, showing the central medullar zone (asterisk) and the surrounding annular zone around. (I,J) Picture of ghosts produced from skeleton contaminated with boring algae or fungi (I), contaminants that were not melted (see Movie 2) and could be recovered separately (J).

In the current literature, most data on organic matrix in corals relates to stony corals (phylum Cnidaria, class Anthozoa, subclass Hexacorallia, order Scleractinia), the main reef-builders, but far less concern other anthozoans belonging to the subclass Octocorallia. Hexacorallia and Octocorallia likely diverged approximately 600 Mya ago (Guzman et al., 2018). Even though octocorals do not form ‘true’ reefs, most of them are still calcifiers: they produce micrometric biominerals called sclerites that are distributed throughout the organism. Many octocorals have a central horny axis composed of sclerotized collagen (like gorgonians), and only some of them, the ‘precious corals’, possess a calcified axial skeleton composed of calcium carbonate and organic matrix (see, e.g. Fig. 1 in Le Roy et al., 2021 for a simplified anthozoan phylogeny and their biominerals, and McFadden et al., 2021 for an extensive phylogeny). Among them, the Mediterranean red coral, *Corallium rubrum* (order Alcyonacea) has ecological, cultural and commercial importance: because of the intense red colour of its skeleton, it has been used for centuries for jewellery and art objects.

With regards to coral organic matrix, most biochemical characterizations apply to proteins. In *C. rubrum*, these proteins have been characterized by electrophoresis (Debreuil et al., 2011a,b) and 102 have been identified by combined transcriptomics and proteomics (Le Roy et al., 2021). Most of these biochemical studies have been performed on the axial skeleton and/or the sclerites (Allemand et al., 1994; Debreuil et al., 2012, 2011a,b; Del Prete et al., 2018a, 2017, 2018b) and serve an important basis for gaining insight into the red coral biomineralization process and more widely, cnidarian biomineralization evolution (Conci et al., 2019; Le Roy et al., 2021; McFadden et al., 2021).

Importantly, the sclerites and the axial skeleton of *C. rubrum* are formed by different specialized isolated cells and epithelium, respectively (Allemand, 1993). Moreover, axial skeleton formation is the result of two separated processes that produce two distinct structures, the central medullar and the annular skeletal regions. The first one - taking place at the apex of coral branches by the aggregation of sclerites initially formed by scleroblasts is responsible for longitudinal branch extension (Allemand, 1993). Then, calcium carbonate and organic matrix are gradually deposited around the medulla by the axial calcifying epithelium and form the annular part, which is responsible for concentric skeleton growth (Allemand, 1993; Debreuil et al., 2012; Perrin et al., 2015; Vielzeuf et al., 2008). When using cryo-grinding of skeletons, the OM associated to the annular or to the medullar regions, are consequently mixed and extracted at the same time: this regrettably precludes identifying the cellular origin (scleroblasts versus axial epithelium) of the OM components and limits the identification and composition of the aggregating material.

With these limitations in mind, we have developed an alternative method that has the potential to improve our understanding of the formation of *C. rubrum* axial skeleton. This method, based on the fact that OM content of *C. rubrum* is high, allows the demineralization of the whole skeleton, a process that keeps the OM ‘in place’, as a coherent and tractable so-called ‘ghost’ Fig. 1 (figures 12 and 13 in Allemand, 1993; figure 2 in Cvejic et al., 2007). In this study, we compare this method to the classical cryo-grinding technique in order to determine the pro and the cons of each protocol.

## RESULTS AND DISCUSSION

**Fig. 2.**
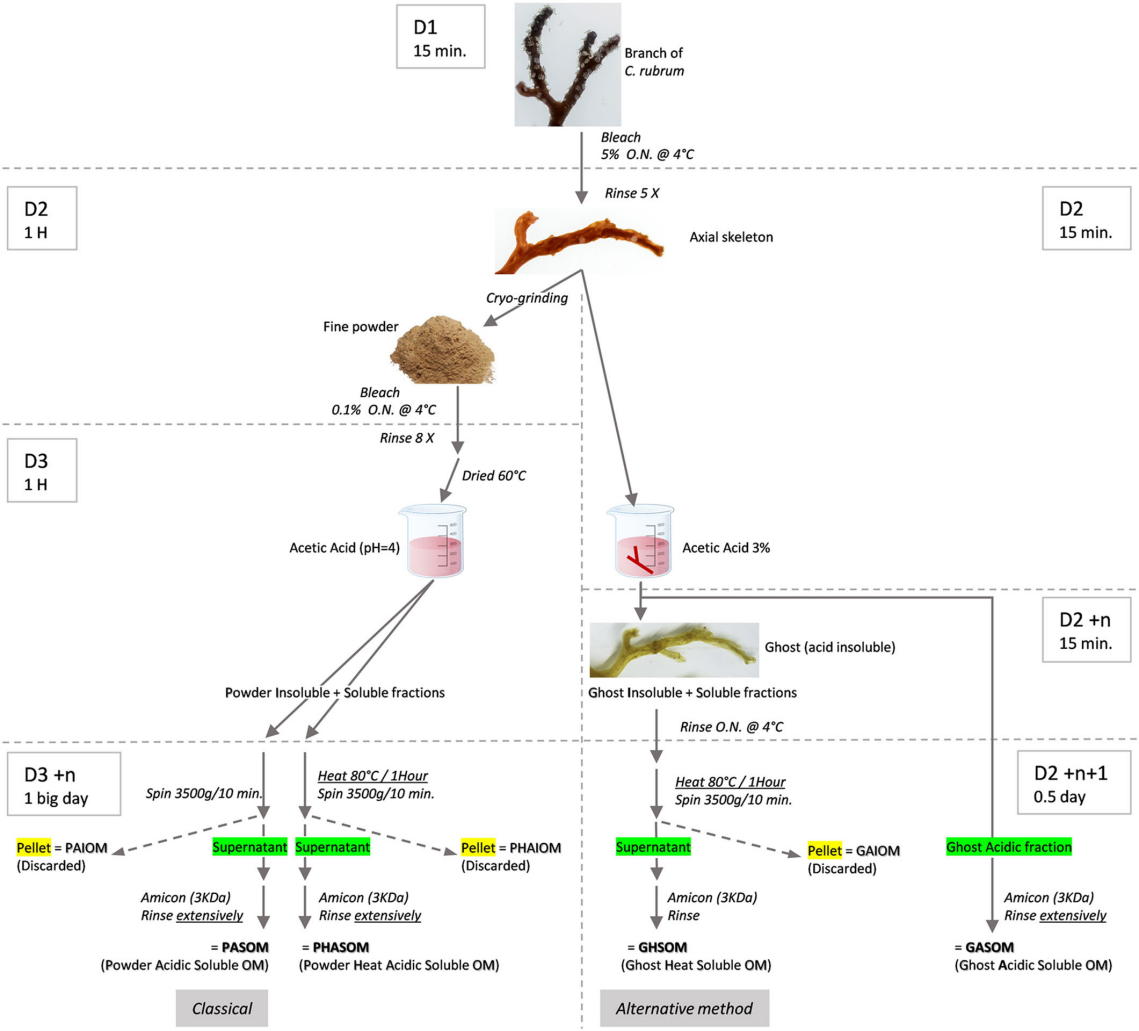
**Outline of the procedures for extracting organic matrix according to a ‘classical’ and an ‘alternative’ protocol.** After bleaching the colony, one red coral skeleton was split into three equal pieces. Two pieces underwent the classical extraction protocol including fine powder grinding followed by acidic demineralization and concentration of the soluble extracts with or without an additional heating step at 80°C after demineralization (PHASOM and PASOM, respectively). The third piece underwent the alternative extraction protocol consisting of demineralization of the axial skeleton (no powder step) leading to the production of an acidic extraction solution (GASOM) and a red-orange ghost. The ghost underwent a melting step (80°C for 1 h) and concentration (GHSOM), see Materials and Methods for details. The timings of the experimental procedures at the different days (D; ‘n’ stands for storage time in the cold room) is given on the sides of the drawing.

As shown in Figs 1 and 2, the main difference between the ‘classical’ and the ‘alternative’ protocols is that, in the second one, the step of cryo-grinding is omitted, which saves time and avoids the use of a cryo-grinder. Indeed, in the alternative protocol, demineralization occurs directly on the cleaned skeleton and allows obtaining an orange coloured ‘ghost’ of skeleton (Fig. 1A-D). Note that decalcification performed on an albinos skeleton gives a non-coloured ghost indicating that ghost colour is due to pigments previously contained into the skeleton and not to a chemical reaction during demineralization (Fig. 1E-G).

The ghost is a tractable jelly-like material that keeps the shape of the skeleton. It is composed of an annular and a medullar zone as clearly visible on Fig. 1G,H. The medullar zone in the centre of the ghost (asterisk in Fig. 1H) corresponds to demineralized material composing the aggregated sclerites, and the surrounding annular zone to the zone of concentric skeleton growth (Allemand, 1993). Though pictures of *C. rubrum* ghosts of skeletons have previously been published (Allemand, 1993; Cvejic et al., 2007), such ghosts were never used for further analyses since this fraction was considered to be insoluble in the demineralizing solution. Our idea was to tentatively solubilize this ‘insoluble’ ghost fraction. We found that heating the ghost at 80°C for 1 h (for a piece of 1 g skeleton) in water made it melt and dissolve (Movie 1). Moreover, we observed that the visible part of the medulla remained un-dissolved. Although we cannot rule out that some components from the medullar zone got dissolved, most of the two visible fractions appeared separated, a property that allows the potential characterisation of the sclerites aggregating material, of yet unknown nature.

In the literature, soluble and insoluble OM fractions (SOM and IOM, respectively) from different calcifying metazoans have often been described as polydisperse molecules, based on their electrophoretic profile on mono-dimensional gels: their migration pattern exhibits a continuum of molecular weights, making a diffuse background despite using different staining techniques (Dauphin, 2006; Marin and Luquet, 2007). In addition, when discrete bands are detectable on a gel, SOMs and IOMs frequently share many of them, suggesting a relative compositional similarity (Gaspard et al., 2007). This finding was confirmed by proteomics performed on IOMs and SOMs of molluscs (Marie et al., 2009), and very recently, on IOMs and SOMS of *C. rubrum*, extracted from both the axial skeleton and the sclerites (Le Roy et al., 2021).

**Fig. 3.**
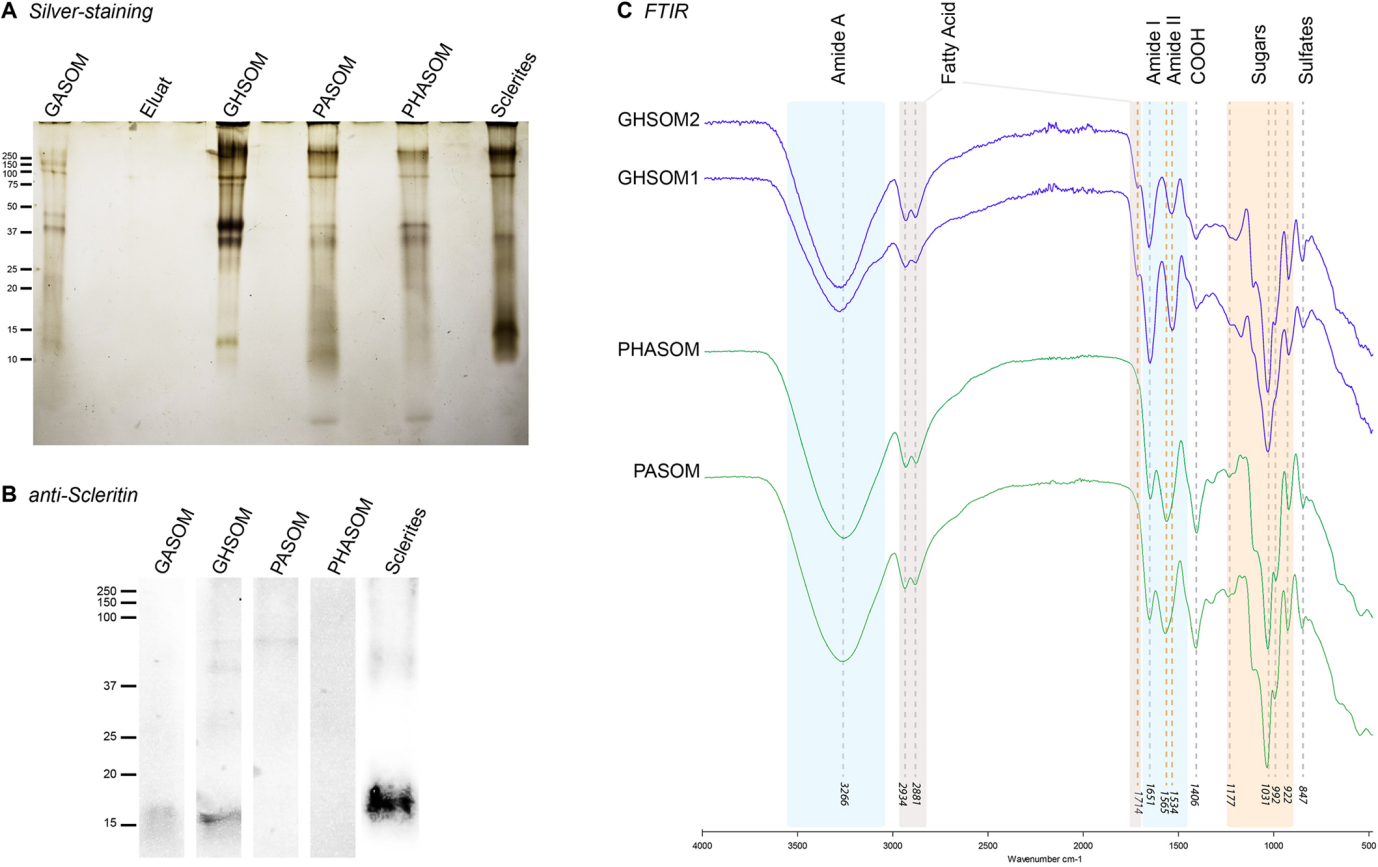
**Extracts analyses by electrophoresis, western blotting and FTIR.** Equivalent quantities of the different extracts (as indicated on the figures) were run on SDS gels, which were further stained with Silver (A), or subjected to western blotting with an anti-scleritin antibody (B). (C) FTIR analyses of the PHASOM, PASOM, and GHSOM (two different extractions) extracts. Transmittance spectra of the different analyses were aligned, and major peak average values are given. Green dashed lines mark conserved peaks, red dashed lines mark peaks that are specific to either the powder or the ghost derived OMs.

In our study we focused on the acidic soluble organic matrix (SOM) and discarded the insoluble fraction, which would need further investigations and testing with different solubilizing agents. Our aim was to compare the composition of the SOM obtained with the classical and alternative protocols. We performed SDS gels electrophoresis with protein (silver stain) and protein+sugar staining (Stains all), western blots and FTIR analyses (Fig. 3; Fig. S1). At first, whichever the analysis conducted, comparison of the SOM between heated and non-heated SOM from the classical protocol (PHASOM versus PHSOM) did not reveal differences, implying that this step (necessary to melt the ghost) had no deleterious effect on the OM quality. Patterns of electrophoretic mobility from SDS gels after silver staining (Fig. 3A) were comparable to the ones published in the previous study of Debreuil et al. (2011a) (in which proteins above 180 kDa were not shown). However, the most discernible bands were observed with the new protocol (melted ghost) (Fig. 3A). Fewer bands were observed with Stains-all (Fig. S1A) than with silver stain, likely due to the different sensitivity of these two stains. However, the profiles of the smears obtained with Stains-all detected compounds of higher molecular weight in the alternative protocol (GHSOM), which suggest an overall better conservation of the molecules or complex of molecules from this protocol.

Western blotting experiments were carried out on the same extracts with two *C. rubrum* custom-made antibodies, one directed against a total soluble skeleton OM extract (anti-crubSOM, Fig. S1B; Debreuil et al., 2011a) and one against the OM protein scleritin (anti-scleritin, Fig. 3B; Debreuil et al., 2012). The anti-crubSOM detected a similar variety of high molecular weight molecules all above 37 kDa in all final extracts, with an additional band around 150 KDa detected in the GHSOM (asterisk in Fig. S1). With the anti-scleritin antibody, the same low molecular weight band (around 15 KDa) was lightly detected in the GHSOM and strongly detected in the sclerites extract. This confirms that only heating the ghost of skeletons makes it possible to reveal low molecular weight components of the axial skeleton SOM.

FTIR spectroscopy (Fig. 3C) was used to qualitatively characterize the presence of functional groups in the GHSOM resulting from the alternative protocol, in comparison to the PASOM and PHASOM extracts from the standard protocol, and to published FTIR work on *C. rubrum* that used a classical extraction protocol (Dauphin, 2006). As a general remark, all spectra of Fig. 3 were superimposable. They clearly showed the presence of characteristic amide A (*ν*_N-H_), amide I (*ν*_C=O_) and amide II (*ν*_C–N_) vibration bands (stretching mode), located at 3266, 1651 and 1565/1534 cm^–1^, respectively. These absorption bands were accompanied by glycosidic stretch bands (1177, 1031 and 922 cm^–1^), resulting from glycosidic linkages (C–O–C). These observations highlight an amino-polysaccharide composition of samples. Interestingly, the narrow band at 1714 cm^–1^ that fuses with the amide I band is located in the region corresponding to the *ν*_C=O_ elongations and suggests the presence of fatty acids. This attribution was consistent with the presence of twin bands attributed to *ν*_C–H_ elongation at 2934 and 2881 cm^–1^. Interestingly, a previous study on the characterization of intra-skeletal organic matrices of Mediterranean corals (Adamiano et al., 2014) mentioned this possibility. The attribution of the band at 847 cm^–1^ is however less assured. Based on comparisons with bibliographical reports (Kizil et al., 2002), this absorption could be assigned to polysaccharides (attributed to C–H and CH_2_ deformation) but might also result from the presence of branched sulphate groups, thus corresponding to C–O–S elongations. Such acidic sulfated sugars were also detected by Dauphin (2006) using X-ray absorption near edge structure (XANES) in addition to FTIR. In the frame of this last hypothesis, this band should be related to the medium absorption observed at 1227 cm^–1^, and which could then correspond to the S=O elongation (Korva et al., 2016). Finally, the absorption band at 1406 cm^−1^ may be attributed either to the delta C-H vibrations (scissoring mode) or to the delta O-H vibration of carboxylic acid function. Conclusively, all the bands characterized in the published FTIR spectrum (Dauphin, 2006) were also present in our present FTIR analyses of the GHSOM implying that the alternative method did not alter the quality of OM.

Overall, our different analyses demonstrate that our alternative method generates similar extracts as the classical method. Furthermore, the heating step at 80°C of the OM ghost is not limiting but rather efficient for gel electrophoresis and western blotting experiments. The ghost represents the acid insoluble fraction of the OM. Although secreted by a cellular epithelium, it has a long and wide shape as the initial skeleton and can be manipulated like a gel. Ghost integrity thus holds by intermolecular interactions, which may result from either covalent bridges or weak interactions between the proteins, sugars and other components of the secreted OM. Our interpretation of the heating treatment at 80°C is that it breaks weak intermolecular interactions (melting, Movie 1) without destroying covalent bonds (electrophoretic pattern shows very limited degradation, Fig. 3 and Fig. S1).

The results discussed above show that our alternative method is both quantitatively and qualitatively satisfactory for organic matrix extraction and biochemical characterization compared to the classical one (summarized in Table 1). Moreover, this method does not require a cryo-grinder and saves time by removing the steps of powder preparation and multiple washings as evidenced when comparing FTIR profiles of PASOM with three final washing versus PASOM and GHSOM with ten and three final washes, respectively (Fig. S2). We have listed in Table 1 the pro and cons of the two methods that we discuss hereafter. Since we did not perform proteomic analysis on the GHSOM extracts, we cannot conclude which protocol contains ‘so-called’ contaminants (Table 1). Three types of contaminants can be accounted for: 1) cytoplasmic contaminants, which may result from moonlighting function of structural components (e.g. actin?) or from cellular debris trapped during calcification; 2) microbes, which could be parasitic contaminants or symbiotic microbiomes; and, 3) macro-organisms such as the boring sponges, which damage and compromise skeleton integrity (Pulido Mantas et al., 2022). We noticed that macroscopic contaminants such as boring organisms were neither dissolved by the acidic decalcification nor melted by the heating step in the alternative protocol (Movie 2; Fig. 1I,J), suggesting that, at least for those contaminants, our alternative protocol may be discriminant (and this should old true for other biominerals). In principle, treatment of these contaminants with 3% acetic acid should not extensively damage their DNA thus allowing DNA extraction and sequencing. Thus, our alternative method also offers a simple procedure to isolate and identify borers of *Corallium* skeleton (or other biominerals). Although beyond the scope of this study, future work should revisit the concept of ‘contaminants’ in biomineralization.

**Table 1.**
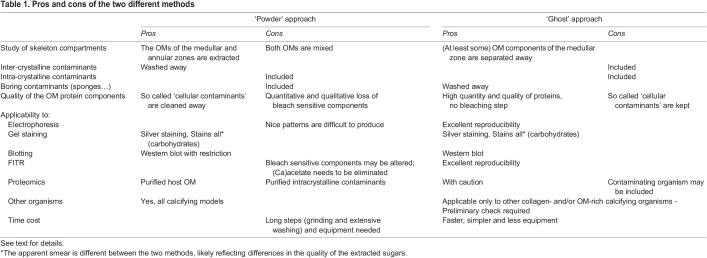
Pros and cons of the two different methods

Regarding future utilization of the OM, immunolabelling with specific antibodies can be performed in western blots but also on fixed and decalcified sections of colonies including their skeletons where the ghost of insoluble organic matrix remains (Debreuil et al., 2011a,b). Such approaches on ghosts will give complementary information on the presence and potential localization of the proteins and other components.

Finally, the ‘classical’ method has been used for a large range of organisms including mollusc shells (Marin et al., 2005), hexacorals and octocorals, whatever their content in organic matrix. We feel that the alternative protocol - and that is one of its limits - can only be applied to skeletal tissues that are enriched in organic matrix, typically skeletons with 1-2% or more of organics, such as the red coral (Allemand et al., 1994). In principle, this protocol is applicable to all other *Corallium* species, but also to other organic-rich calcifiers that are not cnidarians, such as squid or some brachiopods. Importantly, studying diverse biominerals using acidic demineralization and heat treatments was fundamental to the discovery of biominerals' nature (including corals) several centuries ago (Dauphin, 2022). Using the alternative method to perform comparative studies with nowadays technologies will thus complement our understanding of the biomineralization process in precious corals and potentially in other organisms.

## MATERIALS AND METHODS

### Red coral collection and maintenance

Colonies of *Corallium rubrum* were collected in the French Mediterranean Sea near the Marseille coast and reared at the Centre Scientifique de Monaco under constant environmental parameters and daily feeding (see Le Roy et al., 2021).

### Organic matrix extraction

The two protocols, the ‘alternative’ one and the ‘classical’ one derived from (Le Roy et al., 2021) are schematized in Fig. 2.

First experimental steps were common to both: a piece of living coral was bleached with 5% sodium hypochlorite (over-night at 4°C) to obtain a clean axial skeleton devoid of tissues, then rinsed five times with osmosed water, under slow stirring. The clean axial skeleton was split into three equivalent fragments of ∼1 g.

For the classical protocol, two fragments were used: whole skeletons were dried at 60°C and cryo-crushed (Freezer/Mill from Spex SamplePrep) for 5 min to obtain a homogeneous powder with a grain size around 30 µm. The powder was cleaned in 0.1% bleach overnight at 4°C under stirring, then thoroughly rinsed (eight times) with osmosed water and dried at 60°C. It was then suspended in 100 ml of milli-Q water under constant stirring and the pH of the solution was lowered by progressive addition of glacial acetic acid to reach an end-point pH 4 at 4°C until complete demineralisation.

The resulting solution was split in two, one was heated at 80°C for 1 h and the second one, maintained at 4°C. Both were centrifuged at 3500 ***g*** for 10 min, the pellets called Powder Acidic Insoluble Organic matrix (PAIOM) and Powder Heated Acidic Insoluble Organic Matrix (PHAIOM), respectively, were discarded. The supernatants were pre-filtered on a polyethersulfone 0.2 filter, concentrated on a Centricon Plus-70, cut-off 3 kDa (Amicon, Merck KGaA, Darmstadt, Germany) and rinsed ten times with milliQ water. The collected fractions were the Powder Acidic Soluble Organic Matrix (PASOM) and the Powder Heated Acidic Organic Matrix (PHASOM).

In the alternative protocol, the remaining axial skeleton fragments was directly immersed in 500 mL osmosed water, the pH of which was lowered by progressive addition of 15 ml glacial acetic acid (to reach 3%) at 4°C until complete demineralisation (1-2 days). The acidic solution was saved and stored at 4°C to be further processed. The resulting ghost corresponding to the demineralized axial skeleton was transferred with a skimmer into 2 L of Milli-Q water over-night at 4°C, for removing remaining calcium salts. Then, it was transferred in 50 ml milli-Q water and heated at 80°C for 1 h. The resulting melted ghost was vortexed and centrifuged at 3500 ***g*** for 10 min, the pellet named ghost heat insoluble organic matrix (GHIOM) was discarded. This supernatant and the saved acidic solution were then pre-filtered on a polyethersulfone 0.2 filter, concentrated on a Centricon Plus-70, cut-off 3 kDa and rinsed three times with milliQ water. The collected fractions corresponded to the Ghost Heat Soluble Organic Matrix (GHSOM) and the Ghost Acidic Soluble Organic Matrix (GASOM), respectively.

All extracts were aliquoted, flash frozen in liquid nitrogen and stored at −80°C until use.

For the experiment with sclerites, after incubation of a living piece of coral with sodium hypochlorite, the axial skeleton was removed and the bleach solution was centrifuged in order to separate the dissolved tissues (in solution) from the sclerites (according to Debreuil et al., 2011a). Sclerites were then processed like the axial skeleton treated with the alternative protocol.

### FTIR analysis

Fourier Transform Infra-Red (FTIR) spectroscopy was performed on the freeze-dried extracts (minute chips) GHSOM, PASOM, and PHASOM. They were analysed with a Bruker Alpha spectrometer (Bruker Optics Sarl, Marne la Vallée, France) fitted with an Attenuated Total Reflectance (ATR) ALPHA-P device equipped with a mono-reflection diamond crystal in the 4000 **-** 500 cm^−1^ wavenumber range (24 scans at a spectral resolution of 4 cm^−1^). The qualitative assignment of absorption bands was performed manually by comparison with previous spectra descriptions, achieved by our group or available in the literature.

### One dimensional gel electrophoresis and western blots

Electrophoresis of the different fractions of SOM was performed with equal SOM quantity, after denaturation in Laemmli buffer (5 min at 95°C) and running into 12% Criterion™ TGX™ precast gels (Bio-Rad). We used ‘Precision Plus Protein Unstained Standards’ (Bio-Rad, 1610363) as molecular weight markers. Gels were either silver-stained or stained with Stains-all according to (Campbell et al., 1983).

For western blots, we used pre-stained ‘Precision Plus Protein WesternC standard’ (Bio-Rad, 161-0376) as markers. After run, gels were transferred on polyvinylidene fluoride membrane (Trans-Blot1 Turbo™ Midi PVDF Transfer Packs Bio-Rad). Membranes were blocked one hour in TBS-Tween (0.5%) (TBS-Tw), 5% non-fat dry milk, 1% BSA, 1X Carbofree Blocking (SP-5040-125, vector laboratories)] and then incubated with *C. rubrum* specific primary antibodies: either the polyclonal antibody raised against Scleritin (Debreuil et al., 2012) 1:3000 or the polyclonal antibody raised against the soluble organic matrix of axial skeleton (Debreuil et al., 2011a). This incubation step was performed at 4°C overnight in TBS-Tw, 2,5% non-fat milk, 0,5% BSA and 0,5X CFB. After three rinses in [TBS-Tw, 0.5% non-fat dry milk, 0.1% BSA, 0.1X CFB], membranes were incubated for 1 h with the mouse IgG Plus anti-rabbit coupled to Alexa Fluor 680 (1:3000; Invitrogen A32734) in antibody solution, followed by three additional TBS-Tw rinses. The immunoreactive proteins were observed with ChemiDoc MP (Bio-Rad).

## Supplementary Material

10.1242/biolopen.059536_sup1 Supplementary information
