# Supplementary material for: An alternative and effective method for extracting skeletal organic matrix adapted to the red coral Corallium rubrum
Source: Biol Open. 2022 Oct 14;11(10):bio059536. doi: 10.1242/bio.059536 (PMC9581515; doi:10.1242/bio.059536)
Supplement: Supplementary information [file biolopen-11-059536-s1.pdf]

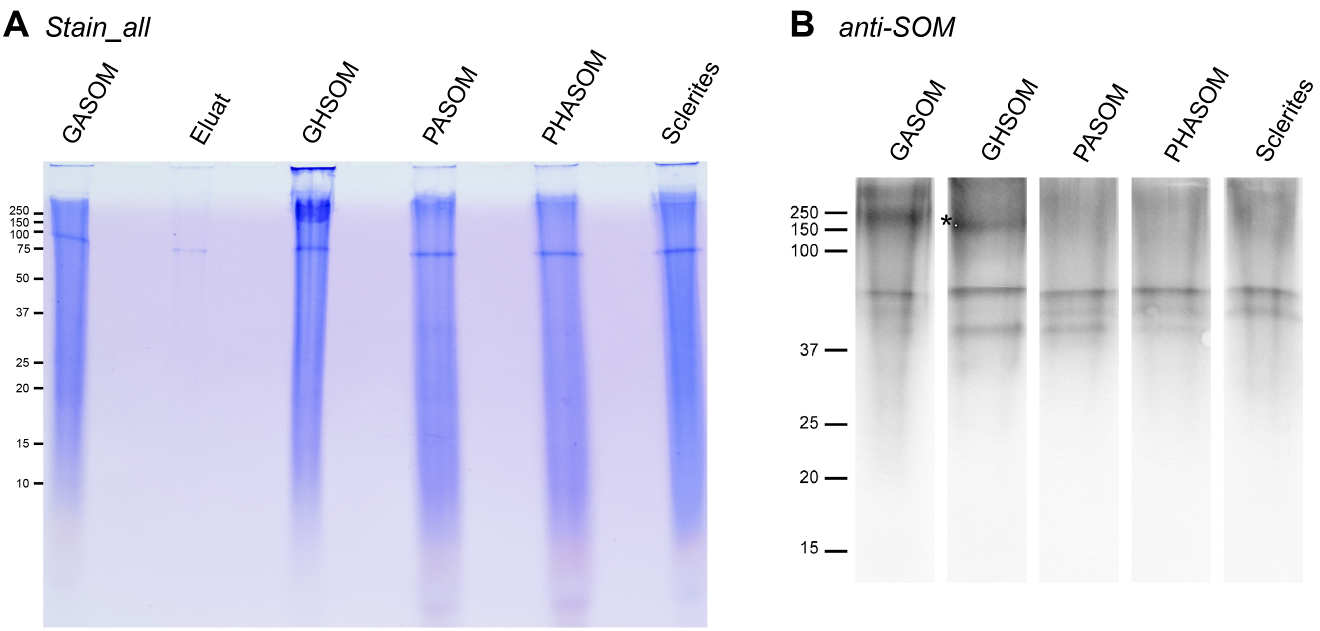

Fig. S1. Extracts analyses by electrophoresis and western blotting (complements Figure 3). The same extracts and gels as in Figure 3: (A) SDS gel stained with the cationic carbocyanine dye “stain-all” showing the global integrity of the extracts; (B) Western blot with an antibody directed against a whole extract of the Organic Matrix (OM) of a *C. rubrum* (Debreuil et al, 2011). Extracts are indicated on top of each lane. The asterisk points to the extra band observed in the GHSOM alternative extraction protocol.

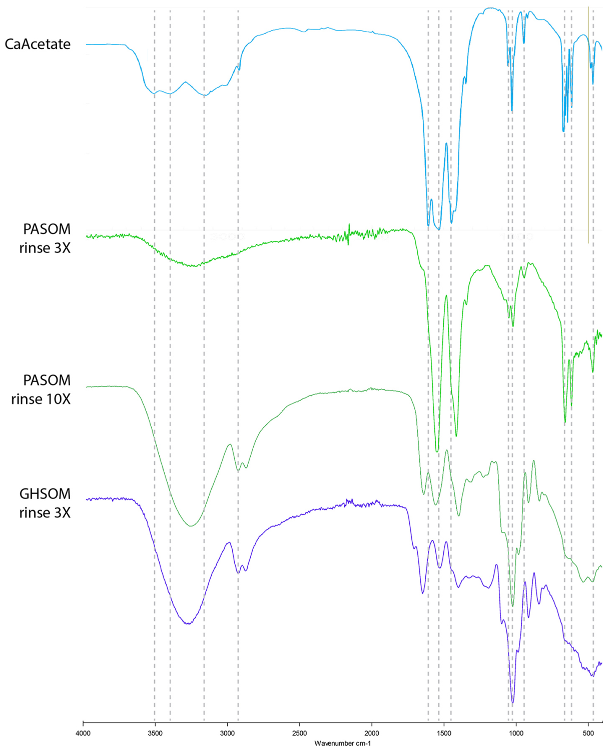

Fig. S2. Effect of multiple rinsing at the last step of powder extraction.

PASOM (classical method) extracts were produced with either 3 or 10 rinsing the last step of the extraction procedure. GHSOM were produced with 3 rinsing. FTIR analyses of these 3 extracts were carried out and spectra were aligned to the reference CaAcetate FTIR spectrum (https://webbook.nist.gov/cgi/cbook.cgi?ID=B6007911&Mask=80).

Note that 3 washes corresponds to the number of washes used for GHSOM (new method) while 10 washes ("multiple rinsing") is the regular, though tedious, number of washes required to clear off the CaAcetate produced during the decalcification of the skeleton powder. Importantly, after only 3 washes, the PASOM extract still contains a high concentration of salts which mask the signal.

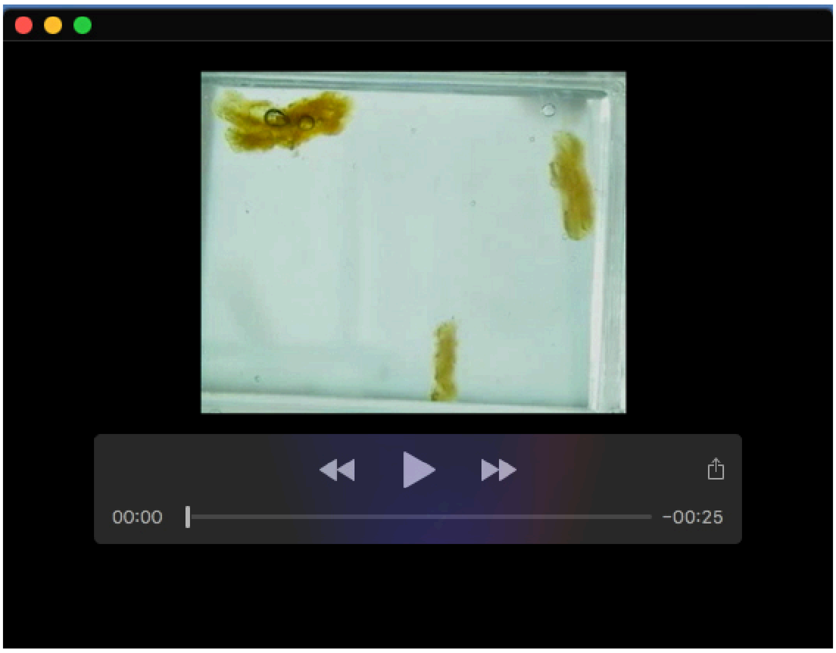

Movie 1. Recording of the melting of the axial skeleton ghost under heat treatment of 1 h at 80°C in osmosed water. The video shows the progressive melting of the annular part of the axial skeleton ghost while the medullar part does not melt.

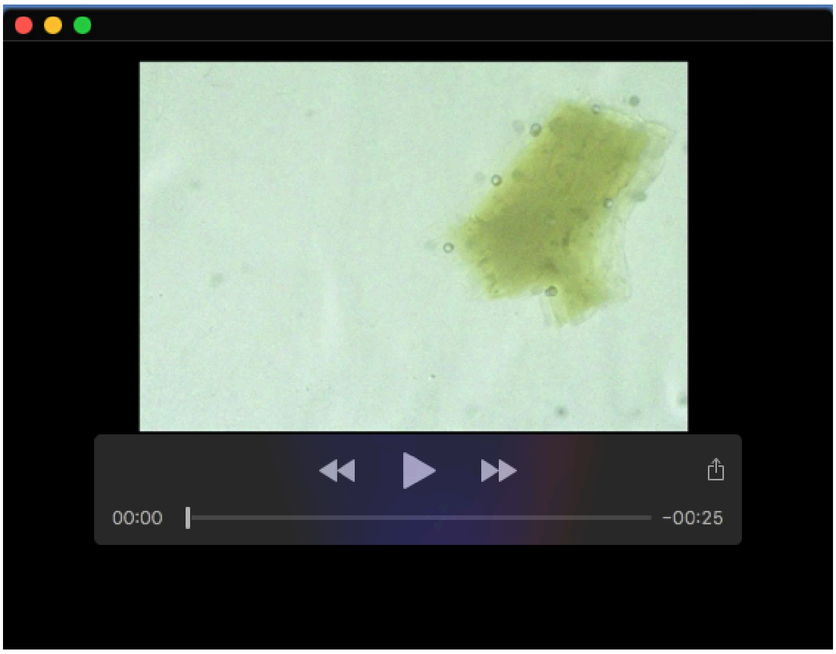

Movie 2. Ghost from a contaminated skeleton. Same as video 1 but with a skeleton invaded with boring organisms, which do not melt.
